# Wheat and Barley Grass Juice Addition to a Plant-Based Feed Improved Growth and Flesh Quality of Common Carp (*Cyprinus carpio*)

**DOI:** 10.3390/ani12081046

**Published:** 2022-04-17

**Authors:** Marian Burducea, Ivayla Dincheva, Lenuta Dirvariu, Eugen Oprea, Valtcho D. Zheljazkov, Cristian-Alin Barbacariu

**Affiliations:** 1 Research and Development Station for Aquaculture and Aquatic Ecology, “Alexandru Ioan Cuza” University, Carol I, 20A, 700505 Iasi, Romania; marian.burducea@uaic.ro (M.B.); dirvariu.lenuta@gmail.com (L.D.); eugen.oprea@uaic.ro (E.O.); alin.barbacariu@uaic.ro (C.-A.B.); 2 Department of Agrobiotechologies, Agrobioinstitute, Agricultural Academy, 8 Dragan Tsankov Blvd., 1164 Sofia, Bulgaria; 3 Crop and Soil Science Department, Oregon State University, 3050 SW Campus Way, 109 Crop Science Building, Corvallis, OR 97331, USA; valtcho.jeliazkov@oregonstate.edu

**Keywords:** phytogenics, inland aquaculture, omnivorous fish, minerals, nutrition, fatty acids

## Abstract

**Simple Summary:**

Several plant extract additives are being increasingly used in aquaculture for their positive effects on fish growth and immunity. Plant extract additives are called phytogenics. The chemical composition of the additives influences their biological activity. The plant extracts used in this study were wheat grass juice and barley grass juice. Their inclusion in a plant-based diet for common carp improved growth performance and flesh quality. The positive effects of the plant extracts could be attributed to their contents of unsaturated fatty acids, essential amino acids, sugars and organic acids.

**Abstract:**

Phytogenics are plant extract additives used for their bioactive properties. The objective of this study was to assess the effect of plant extracts, wheat grass juice (WGJ) and barley grass juices (BGJ) addition to fish diet on growth and meat quality of common carp. Fish (51 ± 33 g initial weight) were fed for four weeks with three plant-based diets: (1) control feed (Con), (2) control feed supplemented with 2% WGJ (Con+WGJ), and (3) control feed supplemented with 2% BGJ (Con+BGJ). The results showed that the inclusion of the two juices in the plant-based feed stimulated the growth and improved meat quality by lowering the fat and ash content. Feed conversion ratio and condition factor were not affected. There were no significant differences in Fe and Zn contents of meat; however, Cu decreased, while Mn was lower in the Con+WGJ group and higher in the Con+BGJ group compared to Con. A high content of unsaturated fatty acids (FA, oleic acid and linoleic acid) and desirable ratios of saturated/unsaturated FA (0.27–0.29) and Ω6/Ω3 (2.5–2.78) were found in all groups. The juices were characterized in terms of lipid profile and polar compounds by GC-MS technique. The observed positive effects can be attributed to the rich composition of juices that included unsaturated FA, amino acids, sugars and organic compounds.

## 1. Introduction

Phytogenics are plant-based feed additives used to promote growth and health in numerous animal species [1]. Their functional roles include improving palatability, stimulating the development of the intestinal microbiota, anti-inflammatory and antimicrobial activity [2]. Lately, the interest in studying the use of phytogenics in aquaculture has increased as such products can counteract or reduce the stress caused by high fish stocking density [3]. According to the latest FAO report (2020), total aquaculture production including fish, algae, ornamental seashells and pearls in 2018 was 114.5 million tonnes with a market value of USD 160.2 billion. Finfish production was 54.3 million tons (USD 139.7 billion) and will continue to grow by up to 32% by 2030 [4]. Technological development and the need to meet market demand are driving aquaculture farmers to increase fish stocking density which can affect fish welfare. At the same time, the high price of fishmeal is motivating farmers to use alternative ingredients. However, plant-based row materials have a lower digestibility than fishmeal, and may contain anti-nutritional factors. This can be a serious problem for carnivorous species as opposed to omnivorous species such as common carp which can readily use plant proteins. Phytogenics are increasingly used in aquaculture due to their bioactive properties [5]. The beneficial effects that phytogenics have on fish depend on the type of extract used, the species from which they are extracted, and the species for which it is used. For instance, essential oils from aromatic plants may improve fish immunity while some other plant extracts may stimulate growth [6,7].

Juices obtained from young cereals such as wheat (*Triticum aestivum* L.) and barley (*Hordeum vulgare* L.) have been used in folk medicine since the early twentieth century. The interest towards these extracts is growing because they are considered healthy and functional foods due to the richness in bioactive compounds such as flavonoids, enzymes, vitamins and minerals [8]. These products are used in detoxification treatments and even in the treatment of diseases such as cancer [9]. Moreover, wheat and barley are globally important crops being cultivated for human or animal consumption. Grass juices are used as superfoods in human food diets [10]. Due to their biochemical composition and proven bioactivity, wheat and barley grass juices are perfect candidates to be tested for phytogenic potential. Previously, we tested the effect of wheat grass juice on some parameters of oxidative stress and blood biochemical profile in common carp feed on a diet that also includes fishmeal. The results showed an improvement in the mentioned parameters using a concentration of 2% of wheat grass juice [11,12]. The aim of this study was: (1) to establish the chemical composition of wheat and barley grass juices by GC-MS, and (2) to test the potential of the juices to be used as phytogenics for common carp fed a vegetable diet by evaluating the growth performance, body composition and FA profile.

## 2. Materials and Methods

### 2.1. Wheat and Barley Grass and Juice Production

Wheat and barley seeds were germinated in plastic trays (30/40 cm) filled with a universal potted flower soil (Florimax, Biofit Exim, Bucharest, Romania) in dark conditions. After three days the seeds germinated and were transferred to natural light; the temperature was 22/25 °C night/day, and watering was completed daily with 1 L of water per tray. The plants were harvested after 10 days by cutting at two centimeters above the growing substrate. The plants were placed in a manual cold press juicer to obtain wheat and barley grass juices (Lexen Healthy Juicer GP27, York, UK).

### 2.2. Nutrient Composition Analyses of Wheat and Barley Grass Juice

The polar (amino and organic acids, carbohydrates) and non-polar metabolites (saturated and unsaturated FA) were determined by gas chromatography–mass spectrometry (GC–MS) according to the method described previously [13]. The GC–MS analysis was carried out on a 7890A gas chromatograph (Agilent Technologies Inc., Santa Clara, San Francisco, CA, USA) interfaced with a 5975C mass selective detector (Agilent). The identification of the components was obtained by comparing the retention times and RI with those of authentic compounds and the spectral data obtained from The Golm Metabolome Database-GMD [14] and National Institute of Standards and Technology (NIST 08) libraries [15].

### 2.3. Fish Trial

All procedures involving animals were conducted in line with the Romanian legislation on the protection of animals used for scientific purposes, approved by the Ethical Commission of Alexandru Ioan Cuza University Decision C3/04.06.2021 (No. 339, 1.02.2022).

The biological material was represented by one summer-old common carp (*Cyprinus carpio* L.) fished from the pond of the Research and Development Station for Aquaculture and Aquatic Ecology at Iasi, Romania. Fish of uniform weight were selected and transferred to a recirculating aquaculture system where they were kept for acclimation for two weeks. The tank working volume was approximately 780 L. Fish stocking density was 0.5 kg/m^3^ (10 fish/tank, mean body weight 51 ± 0.33 g). The duration of the trial was four weeks and the number of replicates was three. The system was equipped with fluorescent tubes to ensure the necessary light (10 h/day). The water parameters were constantly monitored to ensure the welfare of the fish. The mean water parameters were: water temperature 23 ± 0.44 °C; pH 8.3 ± 0.24; dissolved oxygen 8.78 ± 0.31 mg/L (measured with Hach HQ11d portable multiparameter, Hach Company, Loveland, CO, USA); nitrate (NO_3_^−^-N) 39.6 ± 1.53 mg/L; nitrite (NO_2_^−^-N) 0.06 ± 0.00 mg/L; ammonia (NH_3_^+^-N) mg/L not detectable; ammonium (NH_4_^+^-N) mg/L not detectable; phosphate (PO_4_^3−^-P) 1.06 ± 0.01 mg/L; total hardness 10 ± 0.00 German degrees (measured with Hanna Iris HI801 Spectrophotometer and Hanna reagent kits Hanna Instruments, Salaj, Romania).

### 2.4. Experimental Diets

A diet based exclusively on plant-origin ingredients was developed (Table 1). The diet was prepared at the research station by extrusion, grinding and pelletizing of the ingredients into 3 mm pellets. A Bronto EKZ-75 extruder (Cherkasy Elevator Mash Ltd. TM BRONTO, 18018, Cherkasy, Ukraine) was used, power 19 kw/h, capacity for soybean, grains and pea processing 150 kg/h, temperature 115 °C, and pressure 30 atm. This diet was the control feed (Con). The experimental diets consisted of control feed supplemented with 2% wheat grass juice WGJ or barley grass juice BGJ (Con+WGJ and Con+BGJ diets) (*w*/*w*). The total amount of each feed produced was 10 kg. Daily, the feed was weighed and placed in a stainless-steel tray and then sprayed with a hand sprayer with the appropriate amount of WGJ and BGJ and allowed to dry for one hour. Feeding rate was 5% of the body weight and feeding frequency was three times a day. Proximate composition of diets were analyzed with DA 7250 NIR Analyzer (Perten Instruments, Hagersten, Sweden). For each diet, three pooled samples were read three times and the results are presented as means with standard errors.

### 2.5. Fish Growth Parameters

The growth parameters determined in this study were initial body weight (IBW), final body weight (FBW), weight gain (WG), feed conversion factor (FCR), Fulton condition factor (CF), hepatosomatic index (HSS) and viscerosomatic index (VSI). The calculation formulas used are described in [16].

Weight gain (WG, %) = [FBW (g) − IBW (g)]/IBW (g) × 100

Feed conversion factor (FCR, g/g) = feed administrated (feed provided − uneaten feed) (g)/(FBW − IBW) (g)

Fulton’s condition factor (CF) = [FBW (g)/(SL (cm)^3^] × 100,

where SL = standard body length

Hepatosomatic index (HSI) = [liver weight (g)/body weight (g)] × 100;

Viscerosomatic index (VSI) = [visceral weight (g)/body weight (g)] × 100;

### 2.6. Proximate Fish Meat Composition Analysis

DA 7250 NIR Analyzer (Perten Instruments) was used to analyze the biochemical composition of the fish meat. For each variant, three pooled samples were read three times and the results are presented as means with standard errors.

### 2.7. Mineral Elements Analysis of Fish Meat

The analysis of iron, copper, manganese and zinc was performed at National Research and Development Institute for Animal Biology and Nutrition Balotesti (Balotesti, Romania). The concentrations of the chemical elements were determined according to the atomic absorption method described in SR EN ISO 6869: 2002 and STAS 9597/17-86, European Regulation no. 152/2009. In brief, the sample was disaggregated under pressure using a microwave oven (8 min: 130 °C, 5 min: 155 °C, 12 min: 170 °C, 800 W); the solution of the sample was aspirated in the flame of an atomic absorption spectrophotometer with double beam and background correction. The radiation absorption was measured at the wavelength specific to the analyzed element; the equipment used was a Thermo SOLAAR M series atomic absorption spectrometer [17].

### 2.8. Fatty Acids (FA) Analysis of Fish Meat

The analysis was performed at the National Research and Development Institute for Animal Biology and Nutrition Balotesti (Balotesti, Romania). Conversion of FA into methyl esters was performed from dried (65 °C) fat sample according to the method described in SR CEN ISO/TS 17764-1: 2008; quantitative determination of FA and expressed in g/100 g total fatty acid methyl esters (FAME) was performed according to the method described in SR CEN ISO/TS 17764-2: 2008 [18].

### 2.9. Statistical Analysis

The WG, FCR, CF, VSI and HSI parameters were square root transformed; then the normal distribution of all data was verified by the Shapiro–Wilk test. Being normally distributed, all data were subjected to analysis of variance. When the differences between the variants were significant, the Tukey (*p* < 0.05) test was used. The software used was SPSS software version 21 (IBM Co., Armonk, NY, USA). The results are presented as means with standard errors [19].

## 3. Results

### 3.1. Proximate Composition of Diets

The experimental diets had a protein content of 27.36–27.59% and a fat content of 7.56–7.63%, and the variations were not statistically significant (Table 2).

### 3.2. Chemical Composition of Wheat and Barley Grass Juice

The profile of non-polar (fatty acids FA) and polar compounds in wheat grass juice (WGJ) and barley grass juice (BGJ) are presented in Supplementary Materials Tables S1 and S2. Of the group of FA, 11 common compounds were identified in both juices, of which 7 compounds are in the category of saturated FA and 4 in the category of unsaturated FA. By comparison, there was a better saturated FA/total unsaturated FA ratio of BGJ (0.83) due to the fact that the content of decanoic and palmitic acids (the main saturated FA) was lower and the content of palmitoleic, linoleic and linolenic acids (the main unsaturated FA) was higher (Supplementary Materials, Table S3). Of the polar metabolites, WGJ contained 42 compounds, 2 compounds more than BGJ (alanine and sucrose). The chemical groups that were identified included amino acids, organic acids and sugars. In terms of amino acids, both juices contained 6 essential amino acids (valine, leucine, isoleucine, threonine, phenylalanine and lysine), and 3 conditionally essential amino acids (pyroglutamic acid, gutamine and tyrosine), while regarding the non-essential amino acids, WGJ contained 3 (alanine, aspartic acid and asparagine) and BGJ 2 (aspartic acid and asparagine).

### 3.3. Growth Performance of Fish

The production parameters are shown in Table 3. Overall, the addition of juices resulted in a significantly higher final weight by 10% compared with the control group. This resulted in a significantly higher weight gain by 24% in the Con+WGJ group and by 28% in the Con+BGJ group compared with the Con group. Regarding the FCR, although the values were numerically lower in the Con+WGJ or Con+BGJ groups, the differences were not statistically significant. Additionally, there were no significant differences between groups for condition factor, visceral index and hepatosomatic index (*p* > 0.05).

### 3.4. Proximate Composition of Fish Meat

Proximate meat composition analysis (Table 4) showed an improvement in meat quality in the phytogenic groups. Specifically, the fat content was significantly lower in the Con+WGJ group by 53% and in the Con+BGJ group by 47% compared with the Con group. The ash content also decreased significantly in the Con+WGJ group by 31% and in the Con+BGJ group by 25% compared with the control group. The moisture content increased significantly in the phytogenic groups while the protein and salt content remained unchanged among all the experimental groups.

### 3.5. Elemental Composition of Fish Meat

The elemental composition of fish meat is shown in Table 5. The concentration of Fe and Zn were higher in the phytogenic groups compared with those in the Con group but not significantly different. On the other hand, Cu concentration in fish meat decreased significantly in the Con+WGJ group by 23% and in the Con+BGJ group by 57% compared with the Con group. Additionally, the concentration of Mn was significantly lower in the Con+WGJ group by 22% compared with the Con group.

### 3.6. Fatty Acid Composition of Fish Meat

FA composition in common carp meat is shown in Supplementary Materials Table S4. A total of 30 FA peaks were identified, of which 29 could be attributed to specific compounds. The meat of common carp contains 9 saturated FA (SFA), 5 monounsaturated FA (MUFA) and 15 polyunsaturated FA (PUFA). The major FA were palmitic, stearic (SFA), palmitoleic, oleic (MUFA), and linoleic and α-linolenic (PUFA).

The coupling of the analytical results by groups of FA as SFA, MUFA, PUFA and total unsaturated FA (UFA) is presented in Supplementary Materials Table S5, and the composition in Ω3 and Ω6 as well as their ratio Ω6/Ω3 in Supplementary Materials Table S6. According to the results, the use of phytogenics improved the carp meat quality, causing a lower content of SFA and a higher content of PUFA and UFA, a lower ratio of SFA/UFA and a higher ratio of PUFA/MUFA. The content of Ω3 and Ω6 and the ratio Ω6/Ω3 followed the same trend, so the content of Ω3 and Ω6 was higher while the ratio Ω6/Ω3 was lower in the groups with phytogenics compared with the control group.

## 4. Discussion

### 4.1. Chemical Composition of Wheat and Barley Grass Juices by GC-MS

A major challenge for aquaculture is to reduce the dependence on fishmeal and fish oil in feed production because their prices are very high while production has reached its peak and can no longer grow [20,21]. To this end, plant-based ingredients are a viable solution; however, some drawbacks such as lower digestibility and the presence of anti-nutritional factors such as tannins or antitrypsin remain [22]. In this study, wheat grass juice (WGJ) and barley grass juice (BGJ) were used as phytogenics in common carp fed a plant-based diet. The results confirmed the hypothesis that WGJ and BGJ could contribute to a better use of the plant-based feed. The predominant saturated fatty acids (FA) in the tested juices were palmitic, the predominant monounsaturated FA were palmitoleic and oleic, and linoleic and linolenic the predominant polyunsaturated FA, confirming literature reports [23]. Linoleic acid is a polyunsaturated fatty acid found especially in oilseeds [24]. It is a precursor of arachidonic acid, being implicated in the synthesis of prostaglandins, thromboxanes and leukotrienes hormones. Moreover, linoleic and linolenic acids can be converted into eicosanoids, which are bioactive compounds with a role in ameliorating cardiovascular disease, cancer and inflammation [25]. Among the polar compounds identified in WGJ and BGJ, some are essential nutrients in the development of animal organisms (essential amino acids such as valine, leucine, isoleucine, threonine, phenylalanine and lysine, or sugars such as fructose, galactose and glucose), while other compounds might have a role in improving health, for instance glycerol, which is a polyol with antimicrobial and antiviral properties that is widely used in FDA-approved wound and burn treatments [26].

### 4.2. Effects of Phytogenics on Growth Performance of Fish

In this study the final fish biomass was significantly higher for wheat and barley grass juice groups. In general, previous reports indicated that plant extracts could stimulate the growth parameters of fish [12]. In order to be able to explain these effects on growth and development, it is necessary to know the biochemical composition of the extracts. Plant extracts, such as those used in this study, are rich both in nutrients such as unsaturated FA, and amino acids and sugars that are known to have a stimulating role in animal nutrition [27]. For example, Mauerwerk et al. (2020) [28] in a review article concluded that glycerol, which is one of the major polar compounds in grass juices in this study (10 mg/g dw in WGJ and 15 mg/g dw in BGJ), may partially replace corn in fish feed diets due to an adequate energy level. However, further studies are needed to highlight its effect on reproduction or other physiological stages. The major constituents of the lipid profile in grass juices found in this study, linoleic and linolenic acids, can be used as a substrate in energy production in fish [29]. Although the final weight was significantly higher in the groups fed diets with wheat and barley grass juice phytogenics, there were no significant differences in the FCR, CF, VSI and HSI parameters in this study. The most plausible explanation is that plant-based diets have a lower digestibility and efficiency of nutrient utilization, which results in higher FCR and waste production [30]. The FCR is a very important economic indicator as feed represents over 50% of the total cost of fish production. In general, feed manufacturers use fishmeal and fish oil to achieve the lowest possible FCR. The use of plant extracts can counterbalance this effect in carp, as demonstrated by Mocanu et al. (2018) [31]. Moreover, in a recent review, Reverter et al. (2021) [32], analyzing 1647 observations, of which 1522 were validated by eliminating outliers, showed that both the parameters of growth and efficiency of feed use and those of immunity increased significantly in plant-enriched diets.

In this study, the main positive effect of the administration of grass juices in the carp diet on the composition of the meat was the decrease in the fat content from 5.7% (control) to 3% in the group with BGJ and 2.6% in the group with WGJ. This demonstrated that the addition of juices helps to increase the quality of the meat as the fat content is one of the most important quality indicators for the commercialization of fish meat. The fat content is influenced by the quality of the feed and by the technological factors and varies between 2.7% and 17.6%. Because a high content can affect the taste of meat, some European Union countries sell only fish with a fat content below 10% [33]. Another positive effect is the significantly higher moisture content and significantly lower ash content in the WGJ group, while the rest of the parameters did not vary significantly (protein, collagen and salt).

Regarding the mineral analysis (Zn, Fe, Cu and Mn), the values fall within those reported in the literature with a slight increase in the content of Zn and Fe observed in the phytogenic groups. Zinc and Fe are important minerals that are part of many metalioenzymes with a role in the optimal functioning of the immune system, in detoxifying or preventing anemia. An increase in the Fe and Zn content in carp meat was obtained by including a prebiotic (trans-galactooligosaccharide) in the diet [34].

Fatty acid composition of meat and SFA/UFA, PUFA/MUFA and Ω6/Ω3 ratio in this study falls within range reported in the literature [35] with slightly better values for the phytogenic groups. Over time, there has been an increase in the amount of linoleic acid in the diet of developed countries due to the widespread use of soybean oil [36]. This has led, in the American diet, for example, to an increase in the Ω6/Ω3 ratio from 1:1 to 30:1 because the diet is poorer in n−3 FA such as α-linolenic, eicosapentaenoic, docosapentaenoic and docosahexaenoic. Common carp meat, due to its balanced fatty acid ratio Ω6/Ω3 (2.5–2.78) and profile can be considered a healthy food. Indeed, fish meat is an important source of PUFA (eicosapentaenoic and docosahexaenoic acids) in the human diet. The PUFA content in fish meat depends on the PUFA content in the diet. Thus, if in the past, the main source of PUFA in the diet of aquaculture produced fish was fishmeal and fish oil, nowadays, they are partially replaced with vegetable oils [37]. The dietary requirement for 18:2(n-6) or 18:3(n-3) of carp is about 1% [38]. Although WGJ and BGJ used in this study had a high PUFA content (45.96 and 50.15 mg/g dw, respectively), the inclusion of these juices (2%) in the diet ensured only a small amount of the common carp daily requirement for PUFA. Starting from an initial body weight of 51 g and 5% feeding rate, the carp daily requirement for PUFA is 0.51 g (510 mg) according to [38]; thus, the contribution of juices is about 0.5%. The daily requirement for PUFA is supplemented by the content from the rest of the ingredients used in the diet, sunflower meal, DDGS, soybean, peas, barley, oat, rapeseed, corn and wheat, which according to the literature is 34.6% [39], 45.9% [40], 53.79% [41], 43.87–60.68% [42], 59.17% [43], 45.12% [44], 22–35% [45], 38–58.9% [46] and 32.27–43.41% [47] of the total fatty acids, respectively. However, these values vary depending on various factors such as the cultivar and growing conditions. In general, the stimulating effect of plant extracts on growth and quality or health parameters in fish is confirmed and is determined by the type of material used (powder, alcoholic extract, aqueous or essential oils) and the biochemical composition of the extracts [48]. As Reverter et al. (2021) [32] showed, only a small part of the studies biochemically analyzed the composition of the plants used, e.g., only 22 studies (16%) of the total had a biochemical analysis out of which 6 studies analyzed less than three compounds. In this sense, this study adds value to the field of expertise through the complex chemical characterization of juices used as phytogenics for carp.

## 5. Conclusions

In this study, wheat grass juice (WGJ) and barley grass juice (BGJ) were tested as phytogenics for common carp fed on a plant-based diet. The lipid and polar compound profiles of the two juices were highlighted by GC-MS. Juices had positive effects on some parameters of carp production (final weight and weight gain) but also on flesh quality by decreasing the fat content. Moreover, the flesh of WGJ and BGJ groups had a better SFA/UFA, PUFA/MUFA and Ω6/Ω3 fatty acid ratio.

## Figures and Tables

**Table 1:** Diets composition in % as fed basis.

Parameter	Control	Con+WGJ	Con+BGJ
	%		
Sunflower meal	25	24.5	24.5
DDGS	25	24.5	24.5
Soybean	15	14.7	14.7
Peas	15	14.7	14.7
Barley	4	3.92	3.92
Oat	4	3.92	3.92
Rapeseed	4	3.92	3.92
Corn	4	3.92	3.92
Wheat	4	3.92	3.92
Wheat grass juice	-	2	-
Barley grass juice	-	-	2

DDGS—distiller’s dried grains with solubles. Con—control; WGJ—wheat grass juice; BGJ—barley grass juice.

**Table 2:** Proximate biochemical composition of the experimental plant-based diets for common carp.

Parameter	Con	Con+WGJ	Con+BGJ	Anova *p*-Value
Moisture (%)	9.77 ± 0.04	9.74 ± 0.03	9.71 ± 0.05	0.549
Protein (%)	27.36 ± 0.28	27.38 ± 0.20	27.59 ± 0.18	0.727
Fat (%)	7.57 ± 0.04	7.56 ± 0.03	7.63 ± 0.02	0.237
Fiber (%)	7.08 ± 0.14	6.93 ± 0.20	6.81 ± 0.45	0.822
Starch (%)	19.60 ± 0.26	19.60 ± 0.26	19.60 ± 0.26	1.00
Ash (%)	8.89 ± 0.18	9.00 ± 0.10	8.63 ± 0.20	0.334
Sugar (%)	3.57 ± 0.08	3.69 ± 0.11	3.53 ± 0.12	0.571
Calcium (%)	2.96 ± 0.02	2.81 ± 0.13	2.92 ± 0.03	0.471
Phosphorus (%)	1.09 ± 0.01	1.12 ± 0.04	1.10 ± 0.03	0.681

Con—control; WGJ—wheat grass juice; BGJ—barley grass juice.

**Table 3:** Growth performance of common carp fed with a plant-based diet supplemented with 2% wheat grass juice and barley grass juice.

Parameter	Con	Con+WGJ	Con+BGJ	ANOVA *p*-Value
IBW (g)	51.33 ± 0.88	51.67 ± 0.88	51.00 ± 1.00	0.888
FBW (g)	82.33 ± 1.45 ^b^	90.33 ± 0.33 ^a^	90.67 ± 0.88 ^a^	0.002
WG (%)	60.39 ± 0.23 ^b^	74.93 ± 2.57 ^a^	77.86 ± 2.43 ^a^	0.002
FCR (g/g)	2.48 ± 0.02	2.01 ± 0.12	1.93 ± 0.10	0.915
CF	3.27 ± 0.03	3.20 ± 0.05	3.26 ± 0.01	0.352
VSI	10.41 ± 0.15	10.22 ± 0.06	10.19 ± 0.1	0.369
HSI	5.10 ± 0.13	5.13 ± 0.05	5.11 ± 0.03	0.973

IBW—initial body weight; FBW—final body weight; WG—weight gain; FCR—feed conversion factor; CF—Fulton’s condition factor; VSI—visceral index; HSI—hepatosomatic index. Different lowercase letters represent statistically significant differences according to the Tukey test (*p* < 0.05). Con—control; WGJ—wheat grass juice; BGJ—barley grass juice.

**Table 4:** Proximate common carp meat composition fed with a plant-based diet supplemented with 2% wheat grass juice (WGJ) and barley grass juice (BGJ).

Parameter	Con	Con+WGJ	Con+BGJ	ANOVA *p*-Value
Fat (%)	5.7 ± 0.21 ^a^	2.63 ± 0.09 ^b^	3.0 ± 0.21 ^b^	0.000
Moisture (%)	73.17 ± 0.09 ^c^	75.43 ± 0.23 ^a^	74.23 ± 0.23 ^b^	0.001
Protein (%)	15.13 ± 0.19	15.47 ± 0.37	16.07 ± 0.17	0.105
Collagen (%)	1.43 ± 0.03	1.6 ± 0.21	1.37 ± 0.13	0.535
Salt (%)	0.70 ± 0.12	0.13 ± 0.03	0.47 ± 0.20	0.067
Ash (%)	1.57 ± 0.07 ^a^	1.07 ± 0.15 ^b^	1.17 ± 0.07 ^ab^	0.027

Different lowercase letters represent statistically significant differences according to the Tukey test (*p* ˂ 0.05). Con—control; WGJ—wheat grass juice; BGJ—barley grass juice.

**Table 5:** Elemental composition in common carp meat fed with plant-based diet and 2% wheat grass juice (WGJ) and barley grass juice (BGJ).

Parameter	Con	Con+WGJ	Con+BGJ	ANOVA *p*-Value
Fe (mg/kg)	28.79 ± 0.09	29.61 ± 2.93	35.43 ± 3.07	0.210
Cu (mg/kg)	0.52 ± 0.05	0.40 ± 0.02	0.22 ± 0.01	0.020
Mn (mg/kg)	0.83 ± 0.10	0.64 ± 0.06	0.89 ± 0.01	0.021
Zn (mg/kg)	63.76 ± 2.87	69.49 ± 7.42	78.81 ± 2.41	0.167

Con—control; WGJ—wheat grass juice; BGJ—barley grass juice.

## Data Availability

Not applicable.

## Supplementary Materials

The following supporting information can be downloaded at: https://www.mdpi.com/article/10.3390/ani12081046/s1, Table S1. The lipid profile of wheat grass juice (WGJ) and barley grass juice (BGJ). Table S2. The polar metabolites profile of wheat grass juice (WGJ) and barley grass juice (BGJ). Table S3. Fatty acid content (mg/g, dw) depending on the degree of unsaturation in wheat grass juice (WGJ) and barley grass juice (BGJ). Table S4. Fatty acid profile in common carp meat (g FAME/100 g total FAME) fed with a plant-based diet supplemented with 2% wheat grass juice (WGJ) and barley grass juice (BGJ). Table S5. Fatty acid content (g FAME/100 g total FAME) depending on the degree of unsaturation in common carp meat fed with a plant-based diet supplemented with 2% wheat grass juice (WGJ) and barley grass juice (BGJ). Table S6. The Ω3 and Ω6 fatty acid content (g FAME/100 g total FAME) in common carp meat fed with a plant-based diet supplemented with 2% wheat grass juice (WGJ) and barley grass juice (BGJ).
