# Supplementary material for: Wheat and Barley Grass Juice Addition to a Plant-Based Feed Improved Growth and Flesh Quality of Common Carp (Cyprinus carpio)
Source: Animals (Basel). 2022 Apr 17;12(8):1046. doi: 10.3390/ani12081046 (PMC9031860; doi:10.3390/ani12081046)
Supplement: Supplementary file 1 [file animals-12-01046-s001.zip › animals-1614235-supplementaryed.pdf]

Table S1. The lipid profile of wheat grass juice (WGJ) and barley grass juice (BGJ).

Peak	RT	RI	Compound (mg/g dw)		Type of FA	WGJ	BGJ
1	19.58	1320	decanoic acid methyl ester	C10:0	SFA	10.13 ± 0.66	8.16 ± 0.53
2	22.39	1536	dodecanoic acid methyl ester	C12:0	SFA	6.60 ± 0.43	7.92 ± 0.48
3	24.94	1728	tetradecanoic acid methyl ester	C14:0	SFA	3.09 ± 0.20	3.71 ± 0.24
4	27.09	1904	palmitoleic acid methyl ester	C16:1n7	MUFA	2.35 ± 0.15	2.85 ± 0.19
5	27.23	1925	palmitic acid methyl ester	C16:0	SFA	21.66 ± 1.422	16.91 ± 1.11
6	30.41	2096	linoleic acid methyl ester (Ω6)	C18:2n6	PUFA	3.88 ± 0.25	4.66 ± 0.30
7	30.46	2099	oleic acid methyl ester	C18:1n9	MUFA	0.65 ± 0.04	0.48 ± 0.03
8	30.50	2102	linolenic acid methyl ester (Ω3)	C18:3n3	PUFA	42.08 ± 2.75	45.49 ± 2.98
9	31.02	2111	stearic acid methyl ester	C18:0	SFA	4.20 ± 0.27	3.37 ± 0.22
10	34.08	2328	arachidic acid methyl ester	C20:0	SFA	0.54 ± 0.04	1.02 ± 0.07
11	37.72	2534	behenic acid methyl ester	C22:0	SFA	2.97 ± 0.19	3.76 ± 0.25

RT – retention time, RI – retention index, SFA - saturated fatty acids, MUFA – monounsaturated fatty acids, PUFA – polyunsaturated fatty acids

Table S2. The polar metabolites profile of wheat grass juice (WGJ) and barley grass juice (BGJ).

Peak	RT	RI	Compound (mg/g dw)	WGJ	BGJ
1	4.31	1092	alanine	0.09 ± 0.02	Nd
2	4.43	1207	valine	0.14 ± 0.03	0.11 ± .03
3	4.62	1222	malonic acid	1.50 ± 0.35	1.13 ± 0.026
4	5.05	1240	methylmalonic acid	1.25 ± 0.29	0.94 ± 0.222
5	5.38	1259	4-hydroxybutanoic acid	0.14 ± 0.03	0.10 ± 0.02
6	5.85	1277	leucine	1.81 ± 0.42	1.36 ± 00.32
7	5.94	1290	glycerol	10.85 ± 2.54	15.13 ± 3.54
8	6.19	1297	isoleucine	3.65 ± 0.85	2.74 ± 0.64
9	6.37	1308	succinic acid	4.49 ± 1.05	3.36 ± 0.779
10	6.49	1325	fumaric acid	0.68 ±0.16	0.51 ± 0.12
11	6.64	1356	itaconic acid	1.16 ± 0.27	0.87 ± 0.20
12	6.82	1375	threonine	0.75 ± 0.18	0.56 ± 0.13
13	6.88	1401	n-tetradecane	0.23 ± 0.05	0.17 ± 0.04
14	8.53	1477	malic acid	0.41 ± 0.10	0.31 ± 0.07
15	8.64	1488	threitol	0.96 ± 0.22	0.72 ± 0.17
16	8.74	1493	erythreol	0.22 ± 0.05	0.16 ± 0.04
17	8.95	1502	aspartic acid	2.06 ± 0.48	1.55 ± 0.36
18	9.05	1510	salicylic acid	0.14 ± 0.03	0.11 ± 0.03
19	9.13	1518	pyroglutamic acid	0.34 ± 0.08	0.25 ± 0.06
20	9.25	1550	cinnamic acid	0.15 ± 0.03	0.10 ± 0.02
21	9.44	1601	n-hexadecane	0.31 ± 0.07	0.23 ± 0.05
22	9.69	1626	phenylalanine	0.27 ± 0.06	0.20 ± 0.05
23	9.81	1642	p-hydroxybenzoic acid	0.20 ± 0.05	0.15 ± 0.04
24	9.92	1659	asparagine	0.15 ± 0.04	0.11 ± 0.03
25	10.55	1690	xylitol	2.02 ± 0.47	3.51 ± 0.82
26	10.64	1699	arabitol	0.98 ± 0.23	0.74 ± 0.17
27	11.21	1728	trans-aconitic acid	1.37 ± 0.32	2.03 ± 0.47
28	11.49	1764	gutamine	0.21 ± 0.05	0.16 ± 0.04
29	11.57	1775	vanillic acid	0.28 ± 0.07	0.21 ± 0.05

30	12.01	1850	fructose isomer	3.41 ± 0.80	0.56 ± 0.13
31	12.13	1860	fructose isomer	3.23 ± 0.76	0.42 ± 0.10
32	12.21	1877	syringic acid	0.47 ± 0.11	0.35 ± 0.08
33	12.38	1881	galactose isomer	0.17 ± 0.04	0.13 ± 0.03
34	12.53	1889	glucose isomer	0.20 ± 0.05	0.15 ± 0.03
35	12.84	1897	galactose isomer	0.19 ± 0.04	0.14 ± 0.03
36	12.92	1902	glucose isomer	0.17 ± 0.04	0.12 ± 0.03
37	13.03	1911	lysine	0.25 ± 0.06	0.19 ± 0.04
38	13.18	1930	tyrosine	0.55 ± 0.13	0.41 ± 0.10
39	13.91	1942	glucitol	0.90 ± 0.21	0.67 ± 0.16
40	13.97	1961	dulcitol	9.32 ± 2.18	4.99 ± 1.17
41	16.60	2090	myo-inositol	0.62 ± 0.15	1.47 ± 0.34
42	24.80	2620	alpha-D-Glc-(1,2)-beta-D-Fru (Sucrose)	0.10 ± 0.02	Nd

RT- retention time, RI – retention index, Nd – not detected.

Table S3. Fatty acid content (mg/g, dw) depending on the degree of unsaturation in wheat grass juice (WGJ) and barley grass juice (BGJ).

Extract	SFA	MUFA	PUFA	UFA	SFA / UFA	PUFA / MUFA	Ω6/Ω3
WGJ	49.19	3.00	45.96	48.96	1.00	15.32	0.09
BGJ	44.85	3.33	50.15	53.48	0.83	15.06	0.10

SFA - saturated fatty acids, MUFA – monounsaturated fatty acids, PUFA – polyunsaturated fatty acids, UFA – total unsaturated fatty acids.

Table S4. Fatty acid profile in common carp meat (g FAME/100 g total FAME) fed with a plant based diet supplemented with 2% wheat grass juice and barley grass juice.

No.	Fatty Acid Methyl Esters (FAME)		Type of FA	Con	Con+WGJ	Con+BGJ
1	caproic acid	C6:0	SFA	0.07	0.07	0.06
2	caprilic acid	C8:0	SFA	0.28	0.26	0.25
3	capric acid	C10:0	SFA	0.22	0.22	0.21
4	miristic acid	C14:0	SFA	0.96	0.95	0.91
5	pentadecanoic acid	C15:0	SFA	0.14	0.17	0.16
6	pentadecenoic acid	C15:1	MUFA	0.16	0.13	0.12
7	palmitic acid	C16:0	SFA	16.02	15.47	15.04
8	palmitoleic acid	C16:1	MUFA	3.72	3.27	3.21
9	heptadecanoic acid	C17:0	SFA	0.14	0.07	0.15
10	heptadecenoic acid	C17:1	MUFA	0.17	0.23	0.23
11	stearic acid	C18:0	SFA	4.46	4.31	4.34
12	oleic acid cis	C18:1n9	MUFA	39.78	39.16	39.22
13	linoleic acid cis (Ω6)	C18:2n6	PUFA	21.53	22.48	21.98
14	γ-linolenic acid (Ω6)	C18:3n6	PUFA	0.38	0.39	0.34
15	α-linolenic acid (Ω3)	C18:3n3	PUFA	3.33	3.90	3.43
16	conjugated linoleic acid	C18:2	PUFA	0.17	0.20	0.13
17	octadecatetraenoic acid (Ω3)	C18:4n3	PUFA	1.57	1.82	2.29
18	eicosadienoic acid (Ω6)	C20:2n6	PUFA	0.10	0.11	0.14
19	eicosatrienoic acid (Ω6)	C20:3n6	PUFA	0.62	0.62	0.71
20	eicosatrienoic acid (Ω3)	C20:3n3	PUFA	0.82	0.80	0.80
21	arachidonic acid (Ω6)	C20:4n6	PUFA	1.84	1.81	2.03
22	tricosanoic acid	C23:0	SFA	0.12	0.11	0.09
23	docosadienoic acid (Ω6)	C22:2n6	PUFA	0.12	0.12	0.10

24	eicosapentaenoic acid (Ω3)	C20:5n3	PUFA	0.38	0.41	0.37
25	docosatrienoic acid (Ω6)	C22:3n6	PUFA	0.03	0.00	0.19
26	nervonic acid	C24:1n9	MUFA	0.11	0.17	0.12
27	docosatetraenoic acid (Ω6)	C22:4n6	PUFA	0.24	0.31	0.49
28	docosapentaenoic acid (Ω3)	C22:5n3	PUFA	0.27	0.24	0.23
29	docosahexaenoic acid (Ω3)	C22:6n3	PUFA	2.16	2.13	2.60
30	other FA			0.08	0.09	0.08

SFA - saturated fatty acids, MUFA – monounsaturated fatty acids, PUFA – polyunsaturated fatty acids, Con – control, WGJ – wheat grass juice, BGJ – barley grass juice.

Table S5. Fatty acid content (g FAME/100 g total FAME) depending on the degree of unsaturation in common carp meat fed with a plant based diet supplemented with 2% wheat grass juice and barley grass juice.

Sample	SFA	MUFA	PUFA	UFA	SFA / UFA	PUFA / MUFA
Con	22.42	44.33	32.56	76.88	0.29	0.73
Con+WGJ	21.61	43.35	34.33	77.68	0.28	0.79
Con+BGJ	21.22	43.23	34.76	77.99	0.27	0.80

SFA - saturated fatty acids, MUFA – monounsaturated fatty acids, PUFA – polyunsaturated fatty acids, UFA – total unsaturated fatty acids, Con – control, WGJ – wheat grass juice, BGJ – barley grass juice.

Table S6. The Ω3 and Ω6 fatty acid content (g FAME/100 g total FAME) in common carp meat fed with a plant based diet supplemented with 2% wheat grass juice and barley grass juice.

Sample	Ω3	Ω6	Ω6/Ω3
Con	8.57	23.83	2.78
Con+WGJ	9.29	24.83	2.67
Con+BGJ	9.89	24.74	2.50

Con – control, WGJ – wheat grass juice, BGJ – barley grass juice.
